# DHEA-induced ovarian hyperfibrosis is mediated by TGF-β signaling pathway

**DOI:** 10.1186/s13048-017-0375-7

**Published:** 2018-01-10

**Authors:** Daojuan Wang, Wenqing Wang, Qiao Liang, Xuan He, Yanjie Xia, Shanmei Shen, Hongwei Wang, Qian Gao, Yong Wang

**Affiliations:** 1 State Key Laboratory of Analytacal Chemistry for Life Science & Jiangsu Key Laboratory of Molecular Medicine, Medical school, Nanjing University, Nanjing, 210093 China; 2 Prenatal Diagnosis Center, the First Affiliated Hospital of Zhengzhou University, Zhengzhou, Henan 450052 China; 3 Divisions of Endocrinology, the Affiliated Drum Tower Hospital, Medical School, Nanjing University, Nanjing, 210093 China

**Keywords:** Polycystic ovary syndrome, Ovarian hyperfibrosis, TGF-β, SB431542

## Abstract

**Background:**

The polycystic ovary syndrome (PCOS) is a common metabolic and endocrine disorder with pathological mechanisms remain unclear. The following study investigates the ovarian hyperfibrosis forming via transforming growth factor-β (TGF-β) signaling pathway in Dehydroepiandrosterone (DHEA)- induced polycystic ovary syndrome (PCOS) rat model. We furthermore explored whether TGF-βRI inhibitor (SB431542) decreases ovarian fibrosis by counterbalancing the expression of fibrotic biomarkers.

**Methods:**

Thirty female Sprague-Dawley rats were randomly divided into Blank group (*n* = 6), Oil group (n = 6), and Oil + DHEA-induced model group (n = 6 + 12). The model groups were established by subcutaneous injection of DHEA for 35 consecutive days. The 12 successful model rats were additionally divided in vehicle group (*n* = 6) and SB431542-treated group (n = 6). Vehicle group and SB431542-treated group, served as administration group and were intraperitoneally injected with DMSO and SB431542 for additional 14 consecutive days. Ovarian morphology, fibrin and collagen localization and expression in ovaries were detected using H&E staining, immunohistochemistry and Sirius red staining. The ovarian protein and RNA were examined using Western blot and RT-PCR.

**Results:**

In DHEA-induced ovary in rat, fibrin and collagen had significantly higher levels, while the main fibrosis markers (TGF-β, CTGF, fibronectin, a-SMA) were obviously upregulated. SB431542 significantly reduced the expression of pro-fibrotic molecules (TGF-β, Smad3, Smad2, a-SMA) and increased anti-fibrotic factor MMP2.

**Conclusion:**

TGF-βRI inhibitor (SB431542) inhibits the downstream signaling molecules of TGF-β and upregulates MMP2, which in turn prevent collagen deposition. Moreover, ovarian hyperfibrosis in DHEA-induced PCOS rat model could be improved by TGF-βRI inhibitor (SB431542) restraining the transcription of accelerating fibrosis genes and modulating EMT mediator.

**Electronic supplementary material:**

The online version of this article (10.1186/s13048-017-0375-7) contains supplementary material, which is available to authorized users.

## Background

The polycystic ovary syndrome (PCOS) is a common metabolic and endocrine disorder with pathological mechanisms that are poorly understood [1, 2]. Generally, PCOS affects women of childbearing age, and is accompanied by ovarian dysfunction, infertility, hyperinsulinemia, hyperandrogenism, and insulin resistance (IR) [3–5]. According to recent studies, metabolic disturbances are key factors of PCOS pathophysiology and different fasting regimens can have beneficial effects on ovarian function, androgen excess and infertility in women in PCOS [6–8]. In recent years, advances in modern medicine have positively reflected on the study of organ fibrosis in lung, liver, kidney and pancreas. However, internationally or nationally, ovarian fibrosis has not attracted much attention. The key characteristic of PCOS is hyperandrogenism, which has been associated with ovarian hyperfibrosis [9]. Hughesdon was first to identify shared fibrosis features common in PCOS patients, such as increased collagen deposition, cortex and subcortical matrix thickening [10].

Fibrotic diseases are characterized by excessive scaring due to excessive production, deposition, and contraction of extracellular matrix [11]. Transforming growth factor (TGF-β) has been found to have an important role in various tissues and organs fibrosis [12, 13]. There are multiple mechanisms through which TGF-β can be involved in the fibrosis process. For instance, TGF-β can activate the TGF-β/Smads signaling pathway [14], promote fibroblasts transformation into myofibroblasts [15] and destroy the balance between matrix metalloproteinases (MMPs) and tissue inhibitor of metalloproteinases (TIMPs).

TGF-β/Smads signaling has been shown to have a pivotal role in tissue fibrosis [16, 17]. Upon TGF-β activation, TGF-β receptors propagate signal to the nucleus by phosphorylating smad2 and smad3, which form a complex with smad4. The complexes accumulate in nucleus where they regulate the expression of fibrosis related genes [14, 18].

In addition, the overexpression of TGF-β could induce activation and epithelial-mesenchymal transition (EMT) [19, 20], which facilitate fibronectin synthesis, thus, leading to extracellular matrix (ECM) production [21, 22]. Furthermore, TGF-β could cause the deposition of ECM and inhibit the degradation of ECM, by stimulating the secretion of growth factors that promote fibrosis, such as mesenchymal marker vimentin, and a-smooth muscle actin (a-SMA) [23]. These in turn could increase the differentiation of myofibroblast from fibroblasts, subepithelial myofibroblasts and smooth muscle cells [24, 25]. The maintenance of a stable internal environment in ECM depends on the coordination between MMPs and TIMPs. Once the balance is off, prolonged collagen deposition, eventually causes fibrosis. Although TGF-β occupies the central position in the development of fibrosis, the molecular mechanisms have not been well characterized [26].

SB431542 is potent small molecular inhibitor that selectively inhibits transforming growth factor (TGF-β) type I receptor activin receptor-like kinase (ALK5). Ovarian fibrosis is characterized by abnormal proliferation of fibroblasts and excessive deposition of extracellular matrix (ECM) [27]. The transcription of fibrotic genes is dependent on ALK5/Smad3 signaling [28]. It is still not fully understood if the inhibited expression of ALK5 could lead to decrease in fibrosis-related factors.

The main purpose of the present study was to evaluate the role of TGF-β in ovarian fibrosis in dehydroepiandrosterone (DHEA) -induced PCOS rat model. Moreover, we investigated whether ovarian fibrosis was induced via TGF-β/Smad signaling pathway, and whether it ameliorated EMT. In addition, we wanted to test whether the fibrotic signaling pathway can be inhibited, and if inhibited TGF-βRIexpression would improve the symptoms of polycystic ovaries.

## Methods

### Animals

Thirty female Sprague-Dawley (SD) rats, 21 days old, weighing ~50 g, were obtained from Qinglongshan, inc., Nanjing, China. The animals were housed in specific-pathogen-free (SPF) environment (Jiangsu Key Laboratory of Molecular Medicine) with temperature of 22 ± 1 °C, relative humidity of 50 ± 1%, and a light/dark cycle of 12/12 h. Free access to food and water were provided.

All rats were randomly divided in three groups: Blank group (*n* = 6), Oil groups (n = 6), and Oil + DHEA-induced groups (*n* = 18). The treatment was given once a day, using hypodermic injectioncontaining: equivalent solvent (injectable soybean oil) for the Oil group; 6 mg/100 (g·d) dehydroepiandrosterone (DHEA, diluted in 0.2 ml injectable soybean oil) for the Oil + DHEA-induced groups; and saline solution for the blank group. The treatment lasted for 35 days [13, 29]. Eighteen days post- treatment, vaginal smears were collected from all the rats on daily basis at 9:00 am by judging cell types for 14 days, in order to determine their estrous cycles daily. On day 36, all the rats in Blank and Oil-treated groups and six rats of DHEA-induced model groups were euthanized (using intraperitoneal injection of excess 5% chloral hydrate), blood was collected (from the superior vena cava), bilateral ovaries and uteri were dissected. Ovaries were fixed in 4% paraformaldehyde for 24 h at 4 °C, and then embedded in paraffin. The rest of the tissues were frozen in −80 °C for further western blotting and real time- polymerase chain reaction (RT -PCR) analysis. The rest 12 rats from the DHEA-induced model groups were randomized in additional two groups: SB431542 (TGF-βRI Inhibitor) group and vehicle treatment group (control group), six rats per group. During this treatment, DHEA was no longer given to rats. The treatment was done by intraperitoneal injection contaning: 0.2 ml 0.1% DMSO for the vehicle treatment group; 0.2 ml 1uM TGF-βRI Inhibitor (SB431542, S1067, Selleckchem) for SB431542 group (during this treatment, DHEA was no longer given to rats). The treatment lasted for 2 weeks, after which all the animals were euthanized, and blood was collected, and bilateral ovaries and uteri were dissected following the instructions mentioned above.

### Estrous cycle

On day 18 after DHEA treatment, vaginal smears were collected form all rats. Samples were then treated with toluidine blue for 30 min, and consequently cell morphology and estrous cycles were examined under the optical microscope (Leica Microsystems, Germany).

### Serum hormones levels

Blood samples were collected from the superior vena cava. The serum was separated immediately and stored at −20 °C for further hormones determination by enzyme-linked immunosorbent assay (ELISA) (testosterone (T), estradiol (E2), luteinizing hormone (LH), follicle stimulating hormone (FSH)) (rat T, E2, LH and FSH ELISA Kits, USCN, Wuhan, China).

### Haematoxylin- eosin (H&E) and Sirius red tissue staining

Ovaries were embedded in paraffin, and consequently sliced into 4 μm sections. Slices were then fixed in 4% paraformaldehyde for 24 h at 4 °C. Paraffin slices were stained with hematoxylin and eosin in order to examine the tissue morphology under the optical microscope (Leica Microsystems, Germany). Additionally, collagen from ovaries slices was stained by Sirius red to observe the effects of DHEA on rat ovarian fibrosis.

### Immunohistochemistry (IHC)

Four μm tissue sections (ovaries), were first incubated with specific antibodies against TGF-β (3711S, Cell Signaling Technology, CST), P-Smad3 (#9520S, Cell Signaling Technology, CST), a-SMA (ab32575, Abcam), and Collagen IV (Abcam), overnight at 4 °C. All used antibodies were diluted to 1:100 before use. The sections were subsequently incubated with a secondary goat anti- rabbit IgG (H + L) HRP (BS13278, Bioworld Technology) at 37 °C for 30 min. Sections were consequently stained with DAB for 5 s, and counterstained with heamatoxylin (Beyotime Biotechnology), and then covered with coverslips, and observed under optical microscope.

### Real time- PCR (RT- PCR)

Ovarian RNA was extracted using TRIZOL (Beyotime Biotechnology) and the cDNA was synthesized using Reverse Transcription Kit (Takara, China) according to manufacturer’s instructions. RT-PCR reactions were performed with ABI Viia 7 system (USA) using SYBR® Green PCR Master Mix (Takara, China). The primer sequences of target genes are shown in Table 1. The critical threshold cycle (CT) value was determined for each reaction, and the relative mRNA contents were calculated as E = 2^-∆∆Ct^. The housekeeping gene, β- actin, was used as an internal control.

**Table 1:** Primer sequences for real-time RT-PCR

Gene	Primer
TGF-β	F: 5’-TACTGCTTCAGCTCCACAGAGA-3′
	R: 5’-CAGACAGAAGTTGGCATGGTAG-3′
CTGF	F: 5’-CATTAAGAAGGGCAAAAAGTGA-3′
	R: 5’-CACACCCCACAGAACTTAGCC-3′
Fibronectin	F: 5’-TGACAACTGCCGTAGACCTGG-3’
	R: 5’-TACTGGTTGTAGGTGTGGCCG-3’
MMP2	F: 5’-CTTTGCAGGAGACAAGTTCTGG-3’
	R: 5’-TTAAGGTGGTGCAGGTATCTGG-3’
MMP9	F: 5’-AAGCCTTGGTGTGGCACGAC-3’
	R: 5’-TGGAAATACGCAGGGTTTGC-3’
β- actin	F: 5’-TCAGGTCATCACTATCGGCAAT-3’
	R: 5’-AAAGAAAGGGTGTAAAACGCA-3’

### Western blotting

Ovarian proteins were extracted using RIPA lysis buffer (Beyotime) containing 1 mM Pierce™ phosphatase inhibitor (#88667,Thermo Scientific) and 0.1% Halt™ Protease Inhibitor Cocktail (#1862209,Thermo Scientific). The extractive was mixed with 5× SDS-PAGE sample buffer (22.5% 1 M Tris-Cl, pH 6.8, 50% Glycerol, 5% SDS, 0.05% Bromophenol blue, 3.856% DTT). Equal amount of proteins was separated by 10% SDS-PAGE and transferred to PVDF membrane (Merck Millipore), and consequently blocked with 5% BSA blocking buffer for 1.5 h. Target bands were incubated with corresponding primary antibodies, anti-TGF-β (CST, USA), anti-Smad3 (CST, USA), anti-P-Smad3 (CST, USA), anti-Smad2 (CST, USA), anti-P-Smad2 (CST, USA), anti-CTGF (CST, USA), anti-Fibronectin (CST, USA), anti-a-SMA (abcam), anti-MMP2 (Bioworld, USA), anti-MMP9 (CST, USA) and anti-GAPDH (Bioworld, USA) at 4 °C overnight. After washing with TBST three times for 5 min each time, target bands were incubated with secondary antibody (Goat anti rabbit IgG (H + L) HRP) (Bioworld, USA) for 1.5 h at room temperature. At the end, protein strips were stained with Immobilon western Chemiluminescent HRP Substrate (A liquid and B liquid at a ratio of 1:1) (MA01821, MILLIPORE, USA) for rational time. GAPDH was used as internal control.

### Statistical analysis

Data were expressed using mean ± standard deviation (S.D.) or standard error of the mean (s.e.m.) from at least three independent experiments. Differences were analyzed using GraphPad Pism 6.07 software. The multiple comparison was done by one-way ANOVA software using Tukey’s post-hoc test. Binary variables were compared with t-test. *P* ≤ 0.05 was considered statistically significant.

## Results

### DHEA-induced PCOS-like rat and ovarian fibrosis

**Fig. 1:**
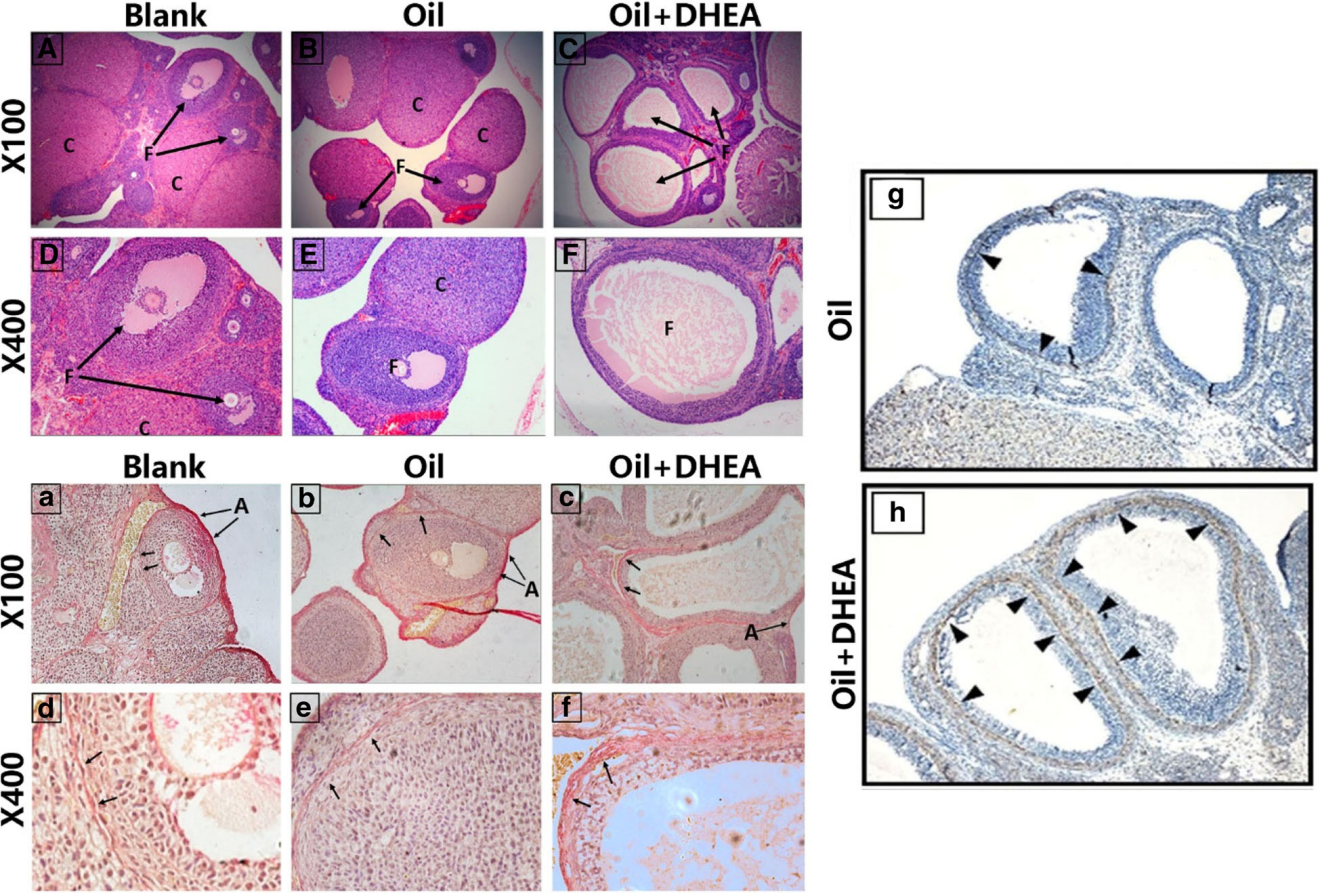
DHEA-induced PCOS-like rat and ovarian fibrosis. Ovarian and follicular morphology of dehydroepiandrosterone (DHEA)-exposed rats and controls, assessed by H&E staining. Normal follicular morphology, thicker granular cell layer and higher number of corpus luteum was observed in blank and oil-treated group compared to Oil + DHEA-treated group. (**A-C**) magnification × 50; (**D-F**) magnification × 100. The detection of ovarian fibrosis. Collagen of ovaries slices was stained by Sirius red to observe the effects of DHEA on rat ovarian fibrosis. (**a**) Blank; (**b**) Oil-treated; **(c)** Oil + DHEA-treated. ×100 (**d-f**) Higher magnification views corresponding to A-C. × 400. Rat ovarian collagen IV detected by immunohistochemical analysis. (**g**) Oil group; (**h**) Oil + DHEA group. ×100. “→” is directed in the direction of fibrosis. “▲” is directed in the direction of collagen IV; C, corpus luteum; F, follicle; A, albuginea

Increased serum hormones levels of T, E2 and LH/FSH were observed in Oil + DHEA-treated rats; however, the change in E2 showed no statistical differences. Interestingly, the LH, FSH and E2/T levels were markedly decreased in Oil + DHEA- group (Table 2 and Additional file 1: Fig. S1). LH and FSH are essential for estrogen production [30]. Under high level LH, the luteinization of theca cells increases [31]. In this study, both LH and FSH levels were significantly reduced. These data suggested that exogenous excessive androgen was not converted to estrogen. The estrous cycle disorder, mainly in the estrus, was observed in the model group (Additional file 1: Fig. S2 A). The mirror images of each period are shown in (Additional file 1: Fig. S2 B). Additionally, ovarian cystic expansion, increased the number of follicles, granular cell layer thinning and the thickening of theca cell layer, while the vast majority of no corpus luteum formation was observed in DHEA-induced rats. Furthermore, cystic follicles were detected in the DHEA group compared to the control (Fig. 1A-F).

**Table 2:** The serum hormone levels in Control and DHEA treated rats

	Blank	Oil	Oil + DHEA
T(ng/ml)	0.488 ± 0.034	0.497 ± 0.051	1.356 ± 0.176 *
E2(pg/ml)	120.137 ± 8.756	119.553 ± 3.485	131.592 ± 7.210
LH(IU/L)	224.737 ± 37.577	217.783 ± 56.983	81.353 ± 15.160*
FSH(ng/ml)	154.58 ± 2.07	73.33 ± 14.8	31.64 ± 11.967**
LH/FSH	1.492 ± 0.313	2.743 ± 0.316	2.958 ± 0.272**
E2/T	0.302 ± 0.030	0.274 ± 0.019	0.128 ± 0.019***

*p ≤ 0.05, **p ≤ 0.01, ***p ≤ 0.02, significantly different from Blank; n = 6 in each group; Data are mean ± SEM

Sirius red staining of ovarian tissues revealed a markedly higher level of fibrin and collagen in Oil + DHEA-induced rat ovaries compared to no-treated and Oil-treated rat ovaries; high level of fibrin and collagen were detected in the follicular basic membrane follicles and interstitial areas (Fig. 1 a-f). Due to the significantly positive expression of fibrosis in DHEA-induced group, we further investigated the collagen IV in ovaries by immunohistochemical analysis (IHC). Our results showed extremely profuse expression in DHEA-treated rat follicular membrane relative to vehicle-treated rats (Fig. 1g and h).

### TGF-β and Smads/p-Smads are up-regulated in oil + DHEA-induced rats

**Fig. 2:**
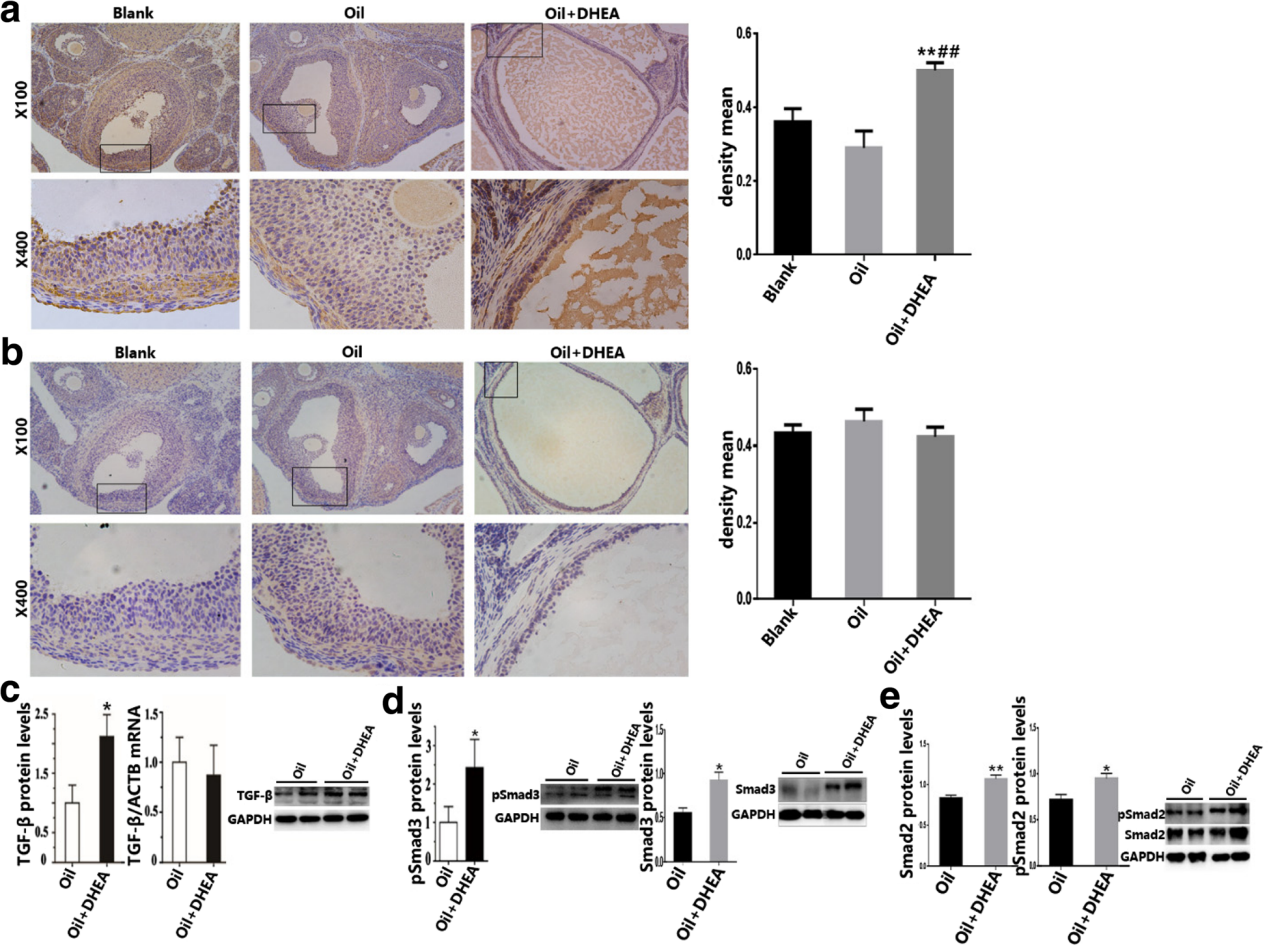
Immunohistochemical analysis of TGF-β and Smads/p-Smads in Oil + DHEA-induced rats ovarian, and the detection of the expression of TGF-β and p-Smad3. **a** Immunohistochemical analysis of TGF-β and the density mean; **b** p-Smad3 and the density mean. **c** Percentage of TGF-β protein in rat ovaries (left) were determined by western blot and quantified by RT-PCR (middle) at least three times. **d** p-Smad3 and Smad3 protein levels. **e** Smad2/p-Smad2 protein levels. *n* = 6 in each group. Mean ± S.D.; *p ≤ 0.05

Based on our previous findings, which revealed higher expression of fibrosis in Oil + DHEA-induced rats, we further investigated whether the TGF-β/Smad3 signaling pathway might be associated with ovarian fibers. TGF-β and p-Smad3 were mainly expressed in theca-interstitial cells and granulosa cells. Briefly, higher expression of TGF-β and p-Smad3 was found in the theca cells, while almost no expression was detected within theca extema cells. Importantly, in Oil + DHEA-induced rats, the levels of TGF-β were significantly more supernal compared to bank control and the oil control group, especially in the granulosa cells. However, according to quantification, we can see that p-Smad3 protein levels have no difference among three groups (Fig. 2a and b). Consequently, we examined TGF-β and p-Smad3 at protein and mRNA level, and detected its expression. Briefly, the expression of TGF-β protein, but not the mRNA, evidently increased in Oil + DHEA-induced rat ovaries (Fig. 2c). Moreover, the expression levels of Smad3 and Smad2 protein in Oil + DHEA-induced rat ovaries were significantly higher compared to Oil-treated group. Similarly, DHEA stimulated Smad2/3 phosphorylation in rat ovaries via TGF-β signaling pathway (Fig. 2d and e).

### Expression of TGF-β downstream signaling molecules and collagen deposition-related protein in DHEA-induced PCOS-like rats

**Fig. 3:**
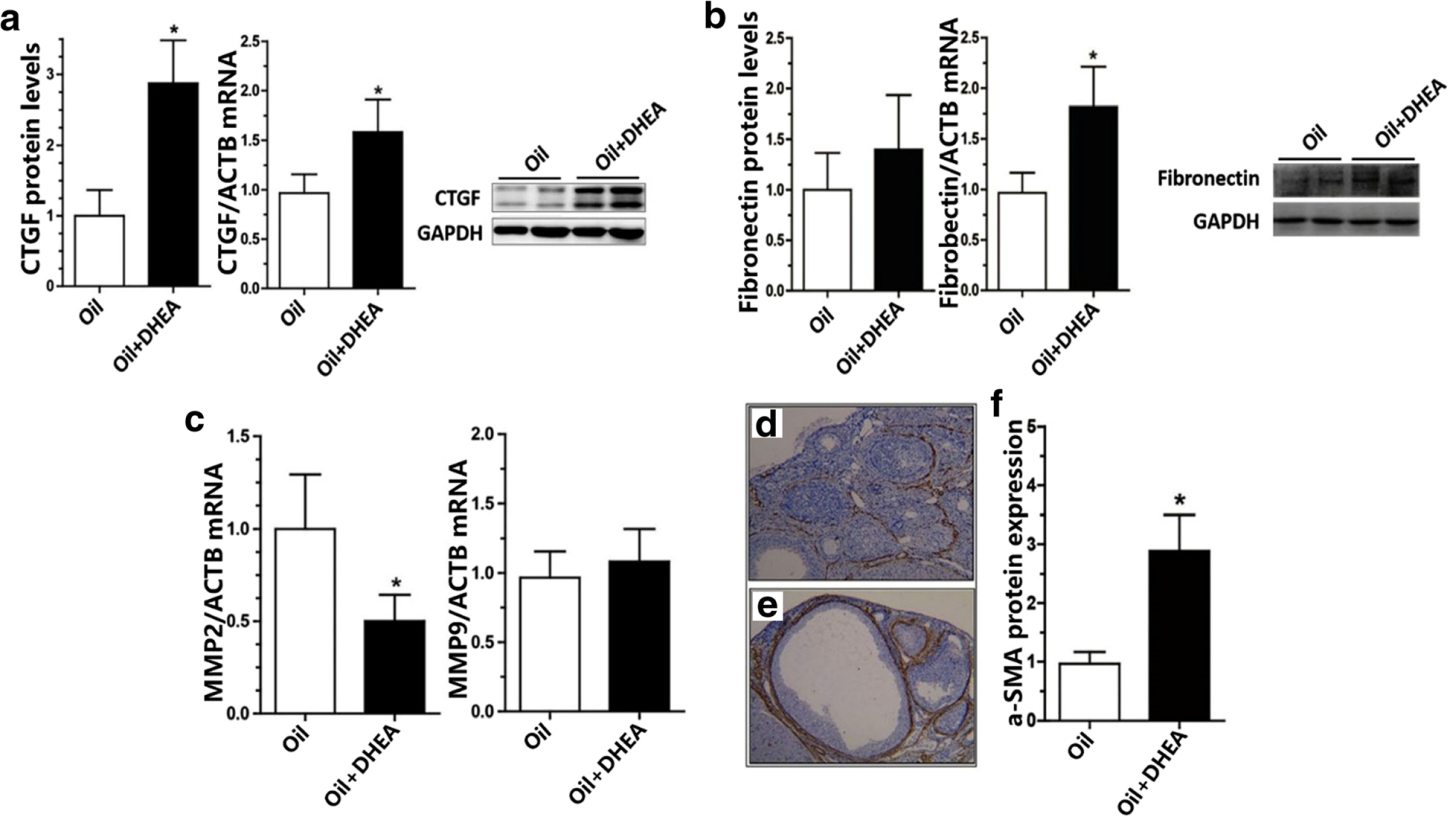
Expression of TGF-β downstream signaling molecules. **a-b** Levels of ovarian CTGF and fibronectin proteins (left) and mRNA (middle), detected by western blotting and quantitative-PCR assay. **c** mRNA levels of MMP2/MMP9 in Oil + DHEA-induced rats compared with Oil-treated rats. a-SMA is highly expressed in DHEA-induced rats. Oil + DHEA-induced rat ovaries (**e**) show a signally proliferation of a-SMA compared with Oil-treated rat ovaries (**d**). ×100. Ovarian protein was harvested to measure a-SMA expression (**f**)**.** *n* = 6 in each group. Data are mean ± S.D.; *p ≤ 0.05

Connective tissue growth factor (CTGF), which is the downstream signaling molecule of TGF-β, interacts with TGF-β to promote ovarian fibrosis [32]. In the DHEA up-regulated rats, both ovarian CTGF and fibronectin protein and mRNA levels were notably enhanced (Fig. 3a and b). In addition, TGF-β can also facilitate extracellular matrix (ECM)-producing cells express a-smooth muscle actin (a-SMA), thus promoting the transformation of fibroblasts into muscle fibroblasts, and therefore leading to ECM over-synthesis and dysregulation of MMPs-TIMPs balance [26]. In Oil + DHEA-induced rats, a-SMA protein showed increasing tendency (Fig. 3d-f). Based on previous data, we detected MMP2 and MMP9 genes expression via RT-PCR analysis; and we observed an obvious down-regulation of MMP2 (Fig. 3c).

### SB431542 improve DHEA-induced ovarian fibrosis

**Fig. 4:**
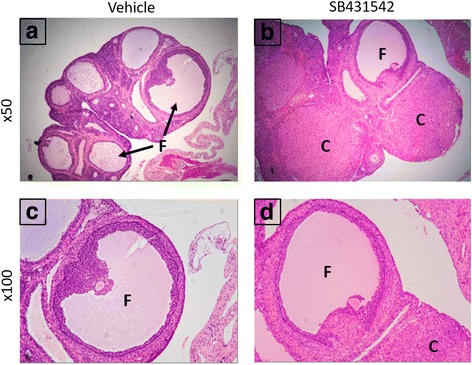
The ovarian morphology changes after SB431542 treatment. Significant increase in number of corpus luteum and significant decrease in cystic follicles of the SB431542-treated group compared to the vehicle group. **a-b** H&E slices, ×50; **c-d** H&E slices, ×100. C = corpus luteum; F = follicle

TGF-β RI is the main receptor of TGF-β, which forms a complex to stimulate smad3 phosphorylation, and consequently promotes target genes transcription. After treating rats with SB431542, no difference in the serum total T was observed compared to the vehicle group; however, significant difference in E2/T levels was observed in the modeling group and the treatment group (Additional file 2: Table S1). These data suggested that body has a strong self-regulating ability. After inhibition of ALK5, the corpus luteum was enhanced and the follicular number was reduced. However, no visible thickening of the granular cell layer was observed (Fig. 4a-d). Moreover, it seemed that the inhibition of TGF-β didn’t have much effect on hormone regulation. The relative hormone levels did not significantly improve after SB431542 treatment. Furthermore, after suspending DHEA treatment, prominent hormone improvement was observed in the vehicle and SB431542 treated group compared to DHEA-induced group (Additional file 2: Table S1 and Additional file 1: Fig. S3), which may be due to innate response ability to directly or indirect effect and adjusted the hormonal levels in the body.

**Fig. 5:**
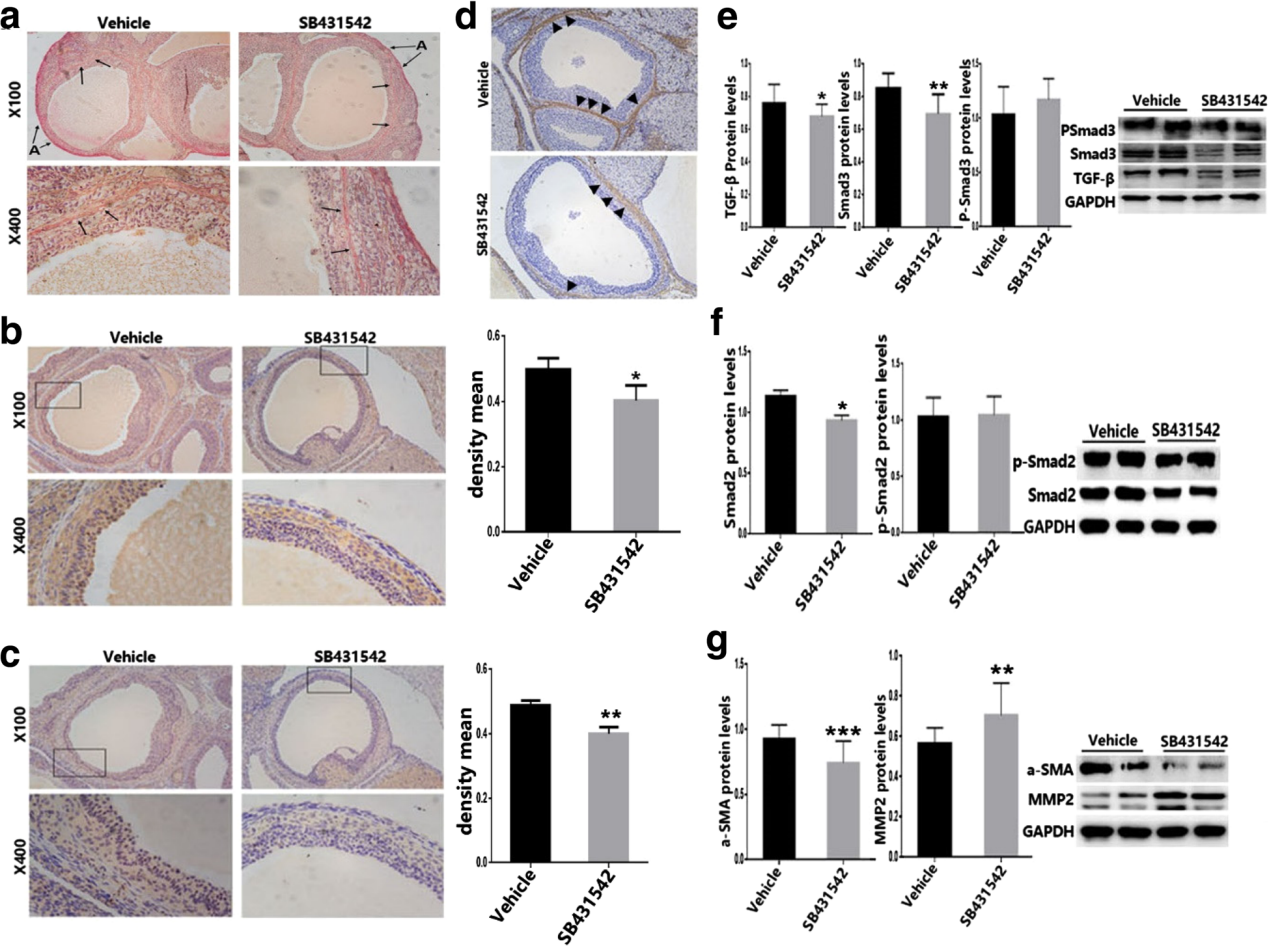
TGF-β signaling pathway was down-regulated after treating with SB431542. **a** Collagen of ovaries slices was stained by Sirius red (Left is Vehicle group, and right is SB431542 treated group). By immunohistochemical analysis, downregulation of TGF-β (**b**) and p-Smad3 (**c**) was observed in SB431542 group compared to vehicle-control rats. **d** Vehicle exposed (the top) and SB431542 treated (the bottom). PCOS-like rat ovary tissue sections were IHC stained for a-SMA. (×100). **e** The expression of vehicle treated and SB431542 treated rat ovarian TGF-β (left) and Smad3 (middle) and p-Smad3 (right) protein were measured relative to GAPDH levels. **f** The expression of vehicle treated and SB431542 treated rat ovarian Smad2 (left) and p-Smad2 (right) protein were measured relative to GAPDH levels. **g** a-SMA and MMP2 protein expression in vehicle and SB431542 treated rat ovaries was tested by Western blot. (n = 6 in each group. The results are presented as mean ± SD of three independent experiments. *p ≤ 0.05, **p ≤ 0.001, ***p ≤ 0.002, “→” is directed in the direction of fibrosis. “▲” is directed in the direction of a-SMA; A, albuginea)

After cutting off the DHEA supply, and consequently treating rats with SB431542, no significant decrease in fibrin and collagen protein expression was observed (Fig. 5a). Interestingly, in model and in blank group (Fig. 1A-F), as well as in the administration group (Fig. 5a), the levels of expression of fibroin in the tunica albuginea ovarii were extremely high. These data suggested that the ovarian albuginea has a high degree of fibrosis and may not be susceptible to medicine. To further examine the correlation between TGF-β/Smad3 signaling pathway and ovarian fibers, we treated rats with SB431542 (TGF-βRI Inhibitor). Briefly, lower expression of TGF-β and Smad3 expressed in granulosa cells was observed in the SB431542 group compared to vehicle-control rats; however, in the theca cells, the level of TGF-β and p-Smad3 did not change prominently (Fig. 5b-c). Moreover, the expression of TGF-β and Smad3 protein were dramatically down-regulated. Nevertheless, it appeared that inhibiting TGF-β to combine with its receptors could not restrain Smad3 phosphorylation (Fig. 5e). Moreover, the TGF-β RI inhibitor led to downregulation of Smad2, but not of p-Smad2 (Fig. 5f).

Additionally, correlation analyses revealed that elevated ovary TGF-β and smad2/3 expression could be associated with ovarian fibrosis, and that blocking of TGF-β/Smad signaling pathway can ease ovarian fibrosis. Furthermore, TGF-βRI Inhibitor can mitigate DHEA exposed ovarian fibrosis by downregulating TGF-β downstream molecular α--SMA (mesenchymal marker vimentin) and upregulating one of ECM-independent factors - MMP2, thus attenuating the accretion of collagen in ovary tissue of vehicle-treated PCOS-like rats (Fig. 5d and g). To sum up, DHEA-induced ovarian fibrosis was mediated by TGF-β signaling pathway. However, despite striking improvements in ovarian function and fibrosis related factors, fibrotic phenotype did not reach effective modification. To conclude, these results suggested that drug could inhibit the excessive production of fibrosis, nevertheless it could not reverse the existing fibrosis.

## Discussion

**Fig. 6:**
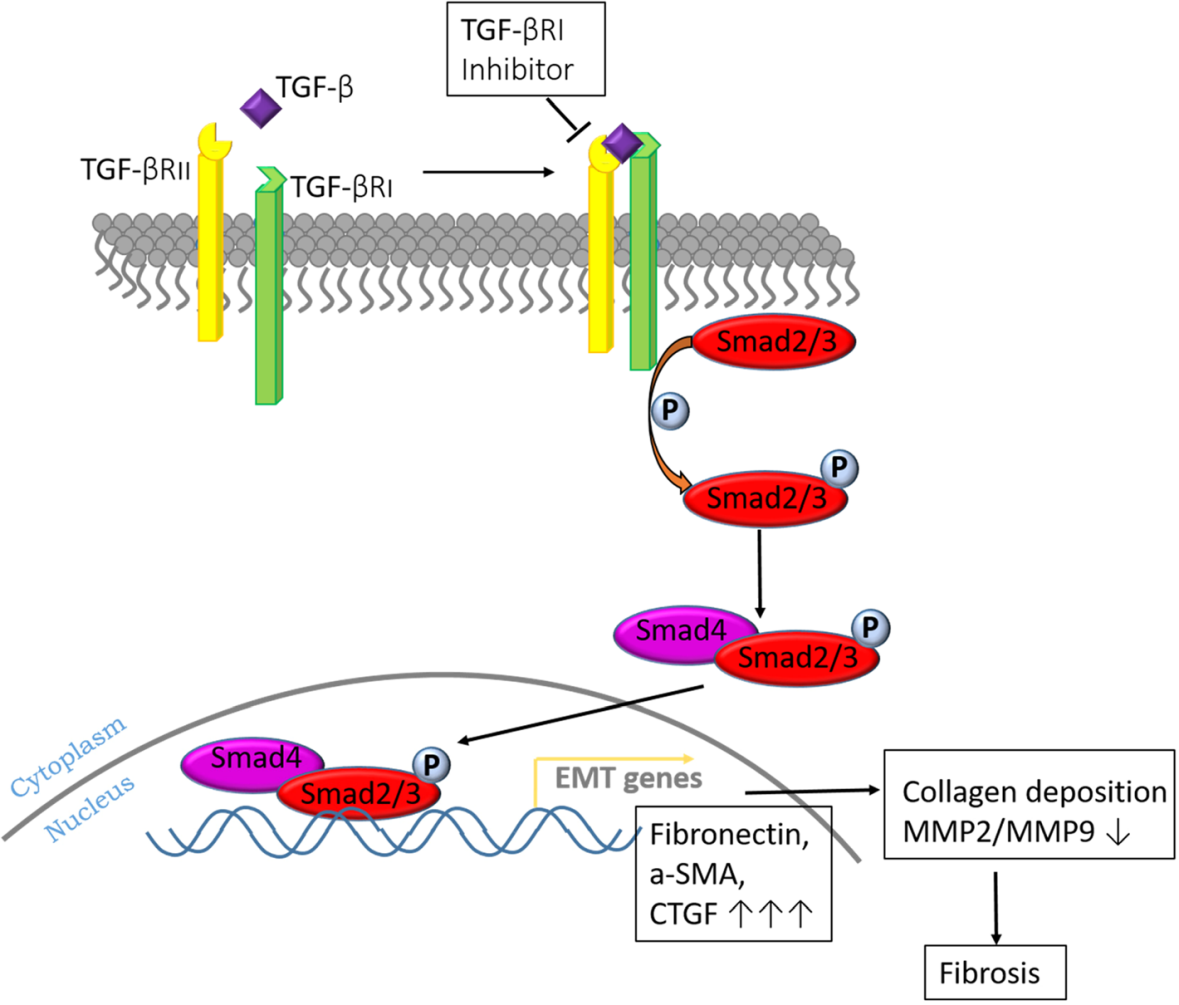
TGF-β-induced fibrosis signaling pathway

In this study, we reported that ovarian fibrosis in DHEA-induced PCOS rats was mediated by TGF-β signaling, while TGF-βRI inhibitors attenuated fibrogenic response by suppressing the elevation of TGF-β and Smad3 (Fig. 6). This caused accumulation of ECM, which promoted ovarian interstitial fibrosis by disrupting the balance between MMPs and TIMPs [33]. A few cystic follicles and a marginal accretion of ovarian granular cell layer were observed in ovaries of SB431542-exposed rat. Accordingly, we showed that ovarian fibrosis in PCOS rat model was formed via TGF-β signaling. Despite the fact that treating PCOS rats with TGF-βRI inhibitors for 2 weeks did not reveal any improvements in Sirius red stained tissue sections following SB431542 exposure, the key factor in fibrosis, TGF-β signaling and its downstream molecular effects all showed a tendency to down-regulate.

Tissue fibrosis is generally considered to arise due to the failure of the normal wound healing or the accumulation of senescence cells [34, 35]. Our results showed that the DHEA-induced PCOS model in rats was a resource of profibrotic signaling observed in ovarian fibrosis (Fig. 1a-f). Ovarian follicular granule cell layer in PCOS rats was thinner compared to normal rats (Fig. 1A-F). It could be that ovarian fibrosis results from the degeneration of granulosa cells, which causes interstitial fibroblast hyperplasia, fibrinogen and collagen deposition. These proteins include profibrotic proteins TGF-β, CTGF and ECM. We believe that TGF-β receptor is an especially important target since TGF-β might activate Smad3 and Smad2. Subsequently, TGF-β/Smads complex might promote CTGF, fibronectin and a-SMA transcription in nucleus.

Based on previous studies, we opted to treat the model rats with TGF-βRI inhibitors (SB531542). Interestingly, our results showed that while the relevant indicators of fibrosis decreased, hormonal levels and fibrotic phenotype showed no significant improvement. The levels of testosterone in PCOS rats (2 weeks post DHEA exposure) dramatically decreased compared to DHEA-induced rats (at the endpoint of 5 weeks post DHEA exposure). The level of LH/FSH in PCOS rats treated with vehicle for 2 weeks, and consequently without using DHEA, differed significantly from DHEA-induced model rats (Additional file 2: Table S1). Thus, we speculate that body has the innate ability to automatically adjust the levels of hormones, following the suspension of the DHEA administration; whereas, inhibiting the binding of TGF-β to the corresponding receptor, does not appear to have any significant role on improving hormonal levels. One of the limitations of the present study relates to the levels of hormones that widely vary among individuals, due to their inconsistency in the estrous cycle. Being an androgen hormone, we hypothesized that DHEA might cause ovarian dysfunction by binding to several nuclear receptors [36], which were possibly independent of TGF-β regulation.

Many recent studies have showed that tissue fibrosis is very hard to reverse, even if targeting certain mechanisms could promote tissue remodeling and tissue function improvement [35, 37], including pulmonary fibrosis and kidney fibrosis [38]. Furthermore, ovaries from SB431542-treated rats didn’t show a reversible appearance compared to vehicle-control rats (Fig. 5a). Nevertheless, even though the fibrotic phenotype did not improve, all the fibrosis biomarkers, such as TGF-β, CTGF and a-SMA were noticeably down-regulated.

Ovarian fibrosis appears challenging but not impossible to reverse. Resveratrol and metformin have shown to be very effective in reversing fibrosis by upregulating phosphorylated AMPK (p-AMPK) expression which attenuates ROS-induced oxidative stress [39–41]. Consequently, in our future studies we plan to address the effect of resveratrol and metformin on ovarian fibrosis upon p-AMPK/ROS signaling pathway in vivo or in vitro.

In the present study we also found that MMP2 was involved in the breakdown of ECM in normal physiological progress [42], which significantly down-regulated at the genetic level in Oil + DHEA-induced rat ovary. Surprisingly, following 2 weeks exposure to SB431542, MMP2 protein showed a marked aggravation. However, MMP9 did not show any significant variations, which implies that MMP2 could have an important role in the development of fibrosis, and that SB431542 has a crucial regulatory effect on MMP2.

Although there are numerous studies on fibrosis, the ovarian fibrosis pathogenesis is still poorly understood. The aim of the present study was to explore whether ovarian fibrosis occurs through TGF-β signaling pathway, and whether the intervention on the TGF-β inhibitor can reverse this phenomenon. Another study limitation is the nature of fibrosis in relation to PCOS, i.e. whether the fibrosis was the cause of PCOS or the result.

## Conclusion

TGF-βRI inhibitor (SB431542) inhibits the downstream signaling molecules of TGF-β and upregulates MMP2, which consequently prevents collagen deposition. Moreover, ovarian hyperfibrosis in DHEA-induced PCOS rat model could be improved by TGF-βRI inhibitor (SB431542) restraining the transcription of accelerating fibrosis genes and modulating EMT mediator. In short, we have demonstrated that DHEA could induce rat ovarian over-fibrosis, which is mediated by TGF-β signaling pathway. Although this hyperfibrosis was hard to reverse, all the fibrotic factors were down-regulated.

## Additional files

Additional file 1: Figure S1. The serum testosterone (T) and *(Estrogen)* E2/T levels in control, DHEA and SB431542-treated rats. *p ≤ 0.05, ***p ≤ 0.002. Data are shown as mean ± SEM. **Figure S2.** *The estrous cycle of DHEA-induced PCOS rats disordered.* **(A)** The estrous cycle of the Blank group, Oil group and Oil + DHEA group rats. The abscissa indicates the age of rats. In the ***proestrus***, many nucleated epithelial cells (NEC) were observed. In the **estrus**, high number of corneous cells (CC) were detected. In the ***metestrus***, visible nucleated epithelial cells, corneous cells and leucocyte (L) were discovered. In the **diestrus**, a mass of leucocyte can be seen in the field of view. **(B)** *Microscopic examination of vaginal smears stained by toluidine blue. × 200.*. D = diestrus; P = proestrus; E = estrus; M = metestrus. **Figure S3.** The hormone levels after SB431542 treatment. **(A)** Serum total T levels; **(B)** E2/T of serum. (DOCX 467 kb)

Additional file 2: Table S1. The serum hormonal levels in Vehicle and SB431542 treated rats. ^∆^*p* ≤ 0.05, ^∆∆^*p* ≤ 0.01, significantly different from Oil + DHEA; ^#^*p* ≤ 0.05, significantly different from Vehicle. Dates are mean ± SEM. (XLSX 11 kb)

## Abbreviations

ALK5: activin receptor-like kinase
a-SMA: a-smooth muscle actin
CTGF: Connective tissue growth factor
E2: Estradiol
ECM: Extracellular matrix
EMT: Pithelial-mesenchymal transition
IHC: Immunohistochemistry
LH: Luteinizing hormone
MMPs: Matrix metalloproteinases
RT- PCR: Real Time- PCR
T: Testosterone
TGF-β: Transforming growth factor
TIMPs: Tissue inhibitor of metalloproteinases
