# Supplementary material for: DHEA-induced ovarian hyperfibrosis is mediated by TGF-β signaling pathway
Source: J Ovarian Res. 2018 Jan 10;11:6. doi: 10.1186/s13048-017-0375-7 (PMC5763573; doi:10.1186/s13048-017-0375-7)
Supplement: Supplementary file 1 — The serum testosterone (T) and (Estrogen) E2/T levels in control, DHEA and SB431542-treated rats. *p ≤ 0.05, ***p ≤ 0.002. Data are shown as mean ± SEM. Figure S2. The estrous cycle of DHEA-induced PCOS rats disordered. (A) The estrous cycle of the Blank group, Oil group and Oil + DHEA group rats. The abscissa indicates the age of rats. In the proestrus, many nucleated epithelial cells (NEC) were observed. In the estrus, high number of corneous cells (CC) were detected. In the metestrus, visible nucleated epithelial cells, corneous cells and leucocyte (L) were discovered. In the diestrus, a mass of leucocyte can be seen in the field of view. (B) Microscopic examination of vaginal smears stained by toluidine blue. × 200.. D = diestrus; P = proestrus; E = estrus; M = metestrus. Figure S3. The hormone levels after SB431542 treatment. (A) Serum total T levels; (B) E2/T of serum. (DOCX 467 kb) [file 13048_2017_375_MOESM1_ESM.docx]

**Additional file 1:**

**
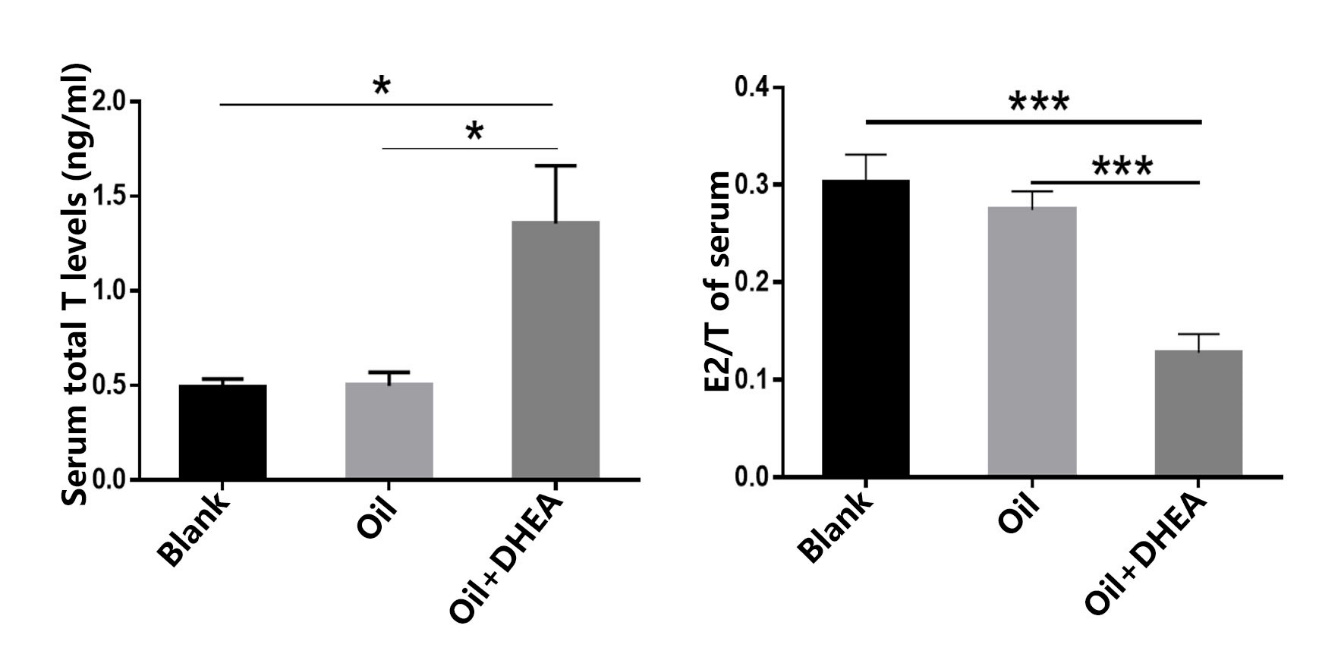
**

**Figure S1.** *The serum testosterone (T) and (Estrogen ) E2/T levels in control, DHEA and SB431542-treated rats*. **p ≤ 0.05, ***p ≤ 0.002. Data are shown as mean ± SEM.*

**
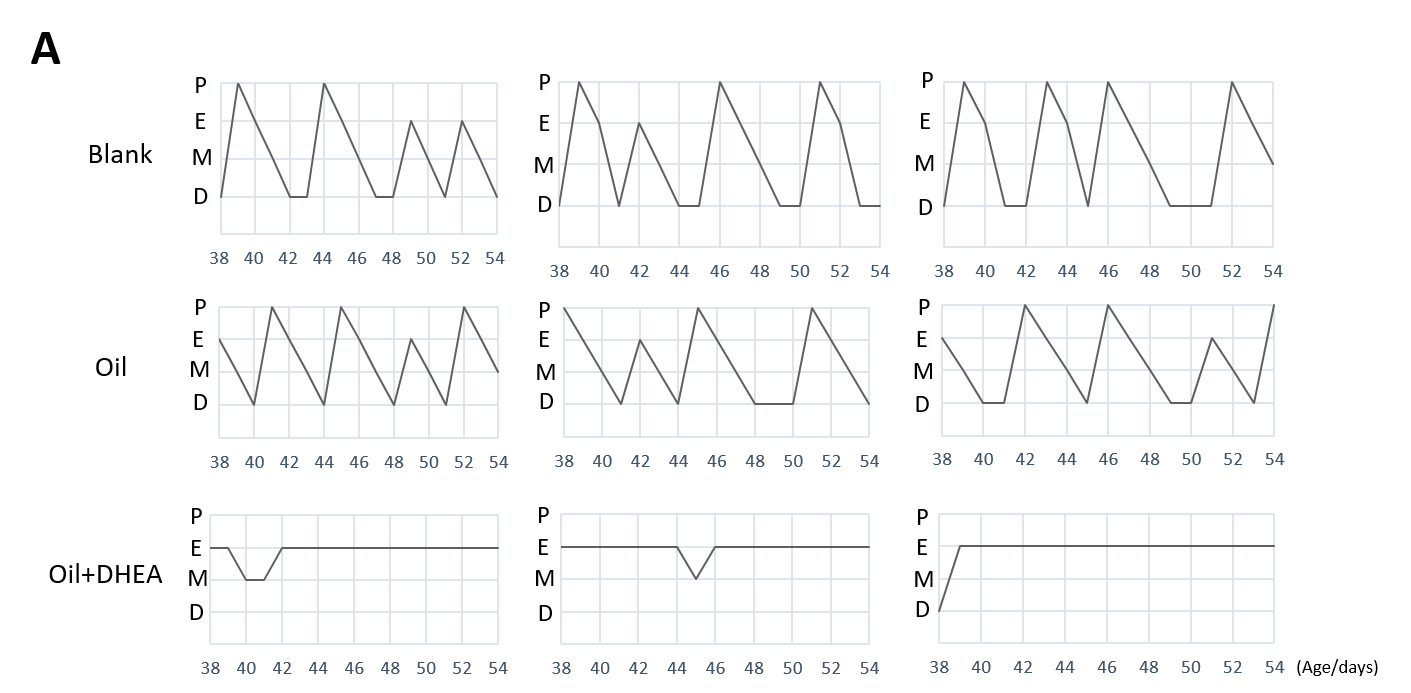

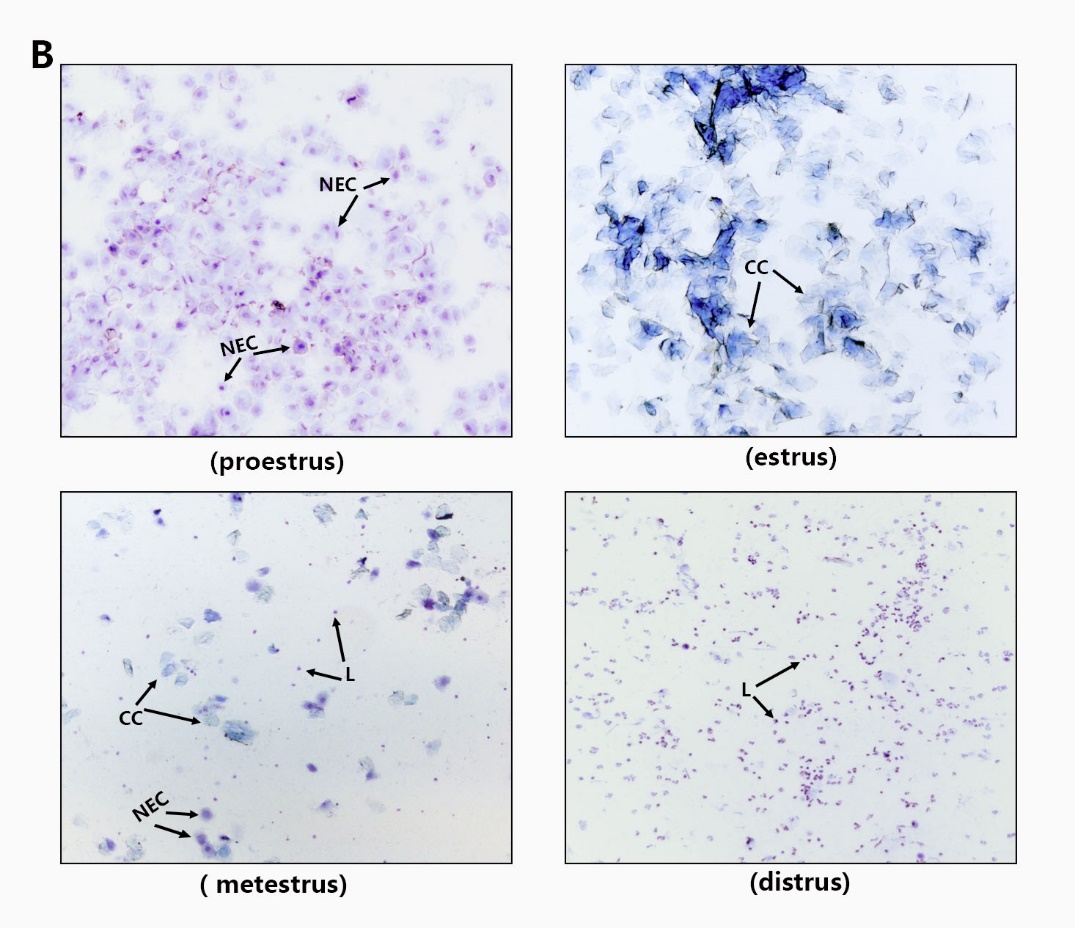
**

**Figure S2.** *The estrous cycle of DHEA-induced PCOS rats disordered. (A) The estrous cycle of the Blank group, Oil group and Oil+DHEA group rats. The abscissa indicates the age of rats. In the* ***proestrus****, many nucleated epithelial cells (****NEC****) were observed. In the* ***estrus****, high number of corneous cells (****CC****) were detected. In the* ***metestrus****, visible nucleated epithelial cells, corneous cells and leucocyte (****L****) were discovered. In the* ***diestrus****, a mass of leucocyte can be seen in the field of view. (B) Microscopic examination of vaginal smears stained by toluidine blue. x 200. D= diestrus; P= proestrus; E= estrus; M= metestrus.*


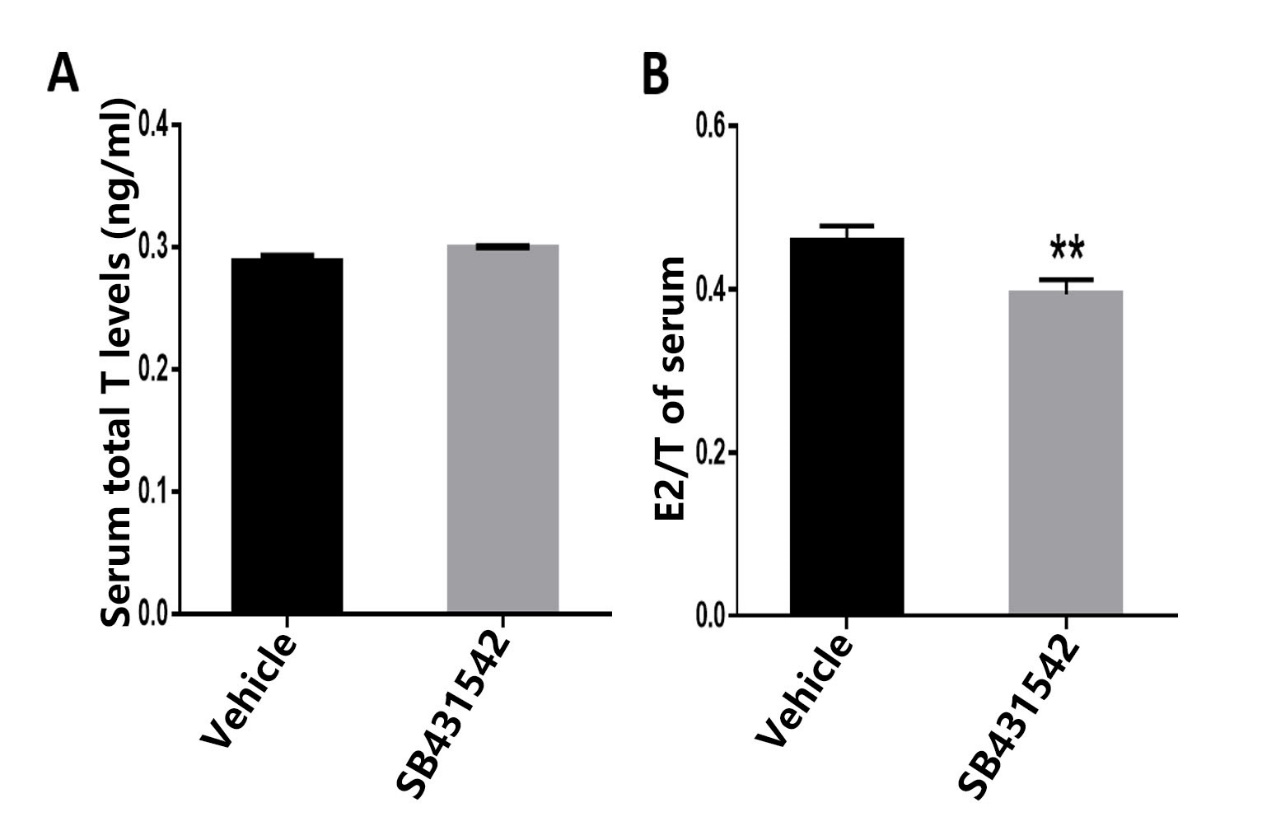


**Figure S3** *The hormone levels after SB431542 treatment.* ***(A)*** *Serum total T levels;* ***(B)*** *E2/T of serum;*
